# Supplementary material for: Tead1a Initiates Transcriptional Priming Through the TEAD1a/YAP‐Notch1‐Spi1/Cebpα Axis to Promote Neutrophil Fate
Source: Adv Sci (Weinh). 2025 Aug 28;12(41):e05441. doi: 10.1002/advs.202505441 (PMC12591127; doi:10.1002/advs.202505441)
Supplement: Supplementary file 4 — Supplementary Dataset [file ADVS-12-e05441-s004.zip › 3-5.zip!/3-5/02-5.cluster/cluster14/cluster14_dotplot.pdf]

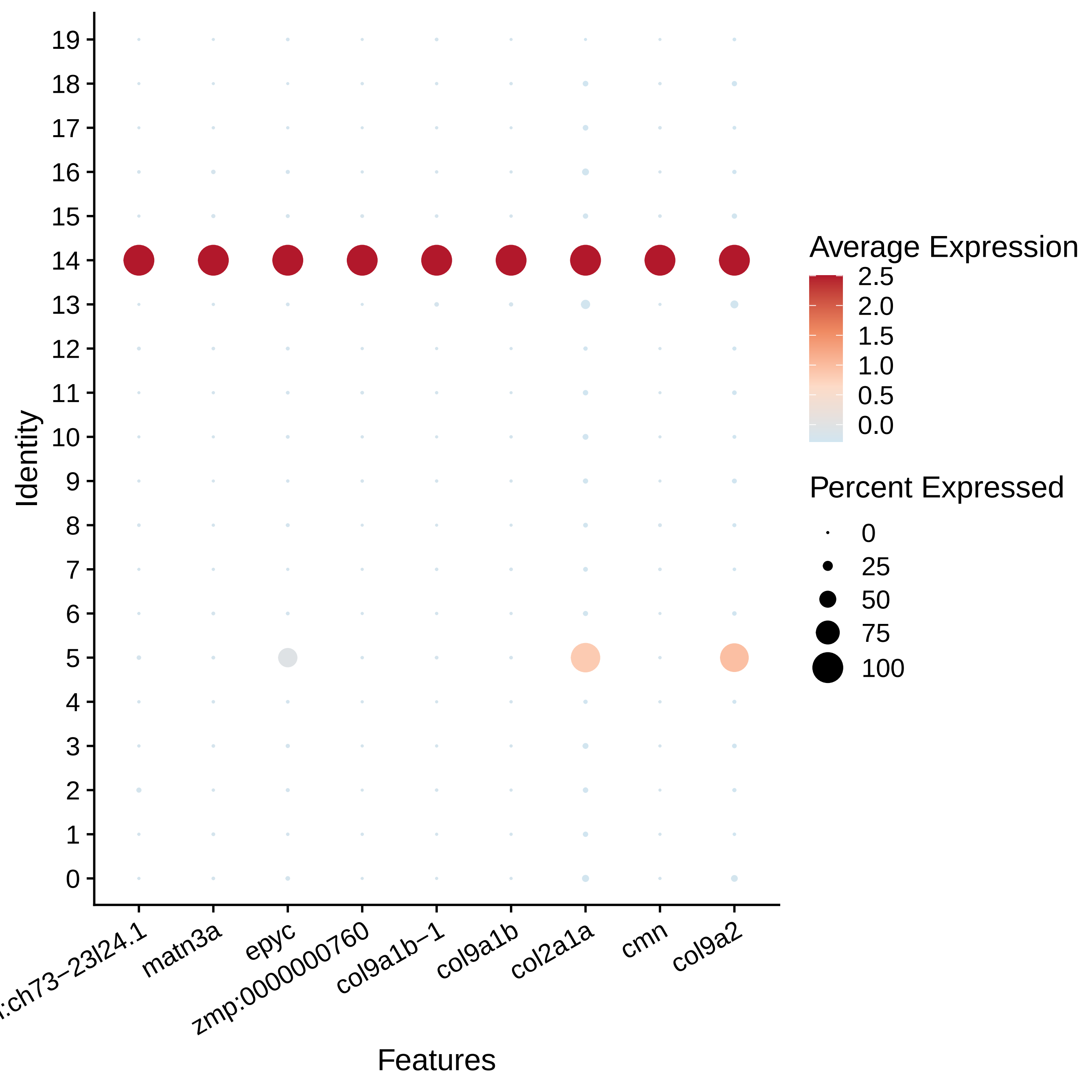
Identity
19
18
17
16
15
14
13
12
11
10
9
8
7
6
5
4
3
2
1
0
:ch73−23l24.1
matn3a
epyc
zmp:0000000760
col9a1b−1
col9a1b
col2a1a
cmn
col9a2
Features
Average Expression
2.5
2.0
1.5
1.0
0.5
0.0
Percent Expressed
0
25
50
75
100
